# A multi-institution evaluation of MLC log files and performance in IMRT delivery

**DOI:** 10.1186/1748-717X-9-176

**Published:** 2014-08-11

**Authors:** James R Kerns, Nathan Childress, Stephen F Kry

**Affiliations:** Department of Radiation Physics, The University of Texas MD Anderson Cancer Center, Houston, TX 77030 USA; UT Houston Graduate School of Biomedical Sciences, Houston, TX 77030 USA; Mobius Medical Systems, LP, Houston, TX 77401 USA; Radiological Physics Center, The University of Texas MD Anderson Cancer Center, Houston, TX 77030 USA

**Keywords:** MLC, MLC error, Dynalogs, RMS error, Tolerance levels

## Abstract

**Background:**

The multileaf collimator (MLC) is a critical component to accurate intensity-modulated radiotherapy (IMRT) delivery. This study examined MLC positional accuracy via MLC logs from Varian machines from six institutions and three delivery techniques to evaluate typical positional accuracy and treatment and mechanical parameters that affect accuracy. Typical accuracy achieved was compared against TG-142 recommendations for MLC performance; more appropriate recommendations are suggested.

**Methods:**

Over 85,000 Varian MLC treatment logs were collected from six institutions and analyzed with FractionCHECK. Data were binned according to institution and treatment type to determine overall root mean square (RMS) and 95^th^ percentile error values, and then to look for correlations between those errors and with mechanical and treatment parameters including mean and maximum leaf speed, gantry angle, beam-on time, mean leaf error, and number of segments.

**Results:**

Results of treatment logs found that leaf RMS error and 95^th^ percentile leaf error were consistent between institutions, but varied by treatment type. The step and shoot technique had very small errors: the mean RMS leaf error was 0.008 mm. For dynamic treatments the mean RMS leaf error was 0.32 mm, while volumetric-modulated arc treatment (VMAT) showed an RMS leaf error of 0.46 mm. Most MLC leaf errors were found to be well below TG-142 recommended tolerances. For the dynamic and VMAT techniques, the mean and maximum leaf speeds were significantly linked to the leaf RMS error. Additionally, for dynamic delivery, the mean leaf error was correlated with RMS error, whereas for VMAT the average gantry speed was correlated. For all treatments, the RMS error and the 95^th^ percentile leaf error were correlated.

**Conclusions:**

Restricting the maximum leaf speed can help improve MLC performance for dynamic and VMAT deliveries. Furthermore, the tolerances of leaf RMS and error counts for all treatment types should be tightened from the TG-142 values to make them more appropriate for clinical performance. Values of 1 mm for the 95^th^ percentile of leaf RMS error and 1.5 mm for the 95^th^ percentile leaf error are suggested as action levels for all treatment types.

## Background

Actual multileaf collimator (MLC) position data can be tracked throughout a treatment delivery in the form of MLC log files. This data is important because it gives a permanent record of the actual MLC positions throughout the course of any intensity-modulated treatment which can be analyzed later. These log files can reveal the extent of MLC positioning errors in specific treatments or used to provide pretreatment quality assurance (QA) [1–4]. If the data of errors is aggregated, the overall MLC performance of a machine or clinic can be determined for comparison or to develop a baseline.

AAPM TG-142 [5] recommendations state that a step and shoot and dynamic MLC test should be run annually to determine the maximum root mean square (RMS) error during delivery, as well as the 95^th^ percentile error of all leaf errors over the course of treatment; the tolerances given for both metrics is 3.5 mm. Given that TG-142 generally uses physical machine performance as the basis for recommendations, this study also studies machine performance to determine appropriate tolerances. Although there have been many studies of MLC log files, a study broadly comparing independent institutions for overall performance has not yet been done. A better understanding of how certain mechanical and treatment planning parameters impact the MLC performance could help physicists develop more robust treatment plans while not unnecessarily limiting planning techniques as well as allow comparison to field-wide standards.

In this work, aggregate MLC data were collected over numerous Varian (Varian Medical Systems, Palo Alto, CA) machines at several institutions for different treatment-delivery modalities to determine the actual MLC performance experienced in clinics. We reviewed tens of thousands of treatment logs to determine typical RMS error values for Varian linacs, compared actual RMS error with TG-142 MLC recommendations, determined whether values varied by institutions and between treatment types, and assessed what parameters contributed to RMS error. With this information, quality assurance tests could be better tailored to catch errors or trends as well as identify clues to understand why certain plans or delivery types have atypical RMS error. This aggregate data could also serve as a benchmark of MLC performance that institutions could use to decide whether treatments should be altered to reduce MLC error values or whether established criteria are too restrictive. In addition, these data could be used to set action levels, indicating when a treatment could be approaching a tolerance limit.

## Materials and methods

### MLC logs

Varian MLC logs used in this study are of the pre-TrueBeam format, known as “dynalog” files, and can be captured on most any Varian linac through the MLC-to-clinac communication terminal. Two ASCII files are generated at the completion of a treatment field delivery, one for the A and B MLC banks. These files contain mechanical information of the linac throughout the treatment delivery, captured in 50 ms intervals. At every instance of information capture, data on MLC actual and planned positions, gantry, jaw, and collimator positions and beam on/hold states are acquired.

Anonymized dynalogs were submitted by six participating institutions. Data files gathered from each institution presented in Table 1 show total files analyzed and the breakdown by treatment type. Most data were of step-and-shoot and dynamic treatments. It should be noted, however, that a step-and-shoot and dynamic treatments typically have many fields and thus many MLC log files per patient. In contrast, only two files (one for each bank) are created for a VMAT delivery, regardless of arc length.

**Table 1:** **Log breakdown summary**

Institution	Total	Step & shoot	Dynamic	VMAT
A	30747	–	30173	574
B	25866	22865	3001	–
C	5624	–	5624	–
D	3850	–	3850	–
E	3211	–	2590	621
F	15912	15912	–	–

Total number of MLC logs are listed by the six institutions as well as by treatment type.

There are numerous pieces of data not recorded by the dynalogs, which limits the number of potential correlations, but this weakness is also an appreciable strength. The treatment planning system and version, linac type, age of the linac, MLC model, treatment site, record and verify system, dose rate, maintenance schedule, and many other parameters are not only not recorded but expected to differ at difference institutions. The drawbacks of this mean the data could be muddled; however, any conclusions and correlations drawn from such data would also be independent of these parameters.

### Data analysis

Examination of dynalog files for various other purposes has been done which also explain some of the terms, but because of the subtle differences in terminology it is helpful to define some of the terms used here. Leaf error is defined as the difference between the MLC (or collimator, gantry, etc) planned position and the actual position. These actual/planned differences are synonymous with error. Leaf RMS error is a single value that is the root mean square error of an individual leaf, taking into account the leaf error at every point over the course of the delivery. The direction of the error does not matter. This value is given in Varian’s Dynalog File Viewer application (DFV) for each leaf. Mean, or bank RMS error is the mean of the leaf RMS errors of a given bank (A or B) and treatment delivery; this is also given in DFV. The 95^th^ percentile error specifically references a single value drawn from the total list of errors captured in the file. The direction of the error does not matter. These data are binned in the DFV, but the exact 95^th^ percentile error value is not immediately available. Mean leaf error is the mean of the error of a given leaf over the course of the delivery, but takes into account the direction, or sign, of the error and is not in the DFV.

MLC RMS error and 95^th^ percentile error data were compared between institutions. For all subsequent analyses, the data were assimilated by delivery type to give the broadest and most representative RMS error values and treatment parameters.

Data were compiled and analyzed using FractionCHECK (Mobius Medical Systems LP, Houston, TX). All mathematical calculations (RMS error, 95^th^ percentile errors, etc.) were done using this software unless otherwise mentioned in the fashion mentioned above. Other parameters like leaf speed were calculated using moving averages by dividing the distance covered by the leaf in 20 capture intervals (20 intervals × 50 ms/interval = 1 s) to give distance per second. This was calculated at every capture interval and the average value was reported as the mean leaf speed. The largest distance per second value was the maximum leaf speed. Only leaves that moved during treatment were included in the analyses. Because treatment type is not recorded in the log file, logs were categorized into treatment type depending on leaf movement (static or dynamic) and gantry movement. As appropriate to the modality, the leaf or bank RMS error was compared to many mechanical parameters including the gantry angle, gantry speed, number of plan segments, number of beam holds during delivery, total beam-on time, and mean and maximum leaf speed during delivery, mean leaf error, and internal tolerance, where applicable. Because of the large amount of data extracted, MATLAB (v7.14, 2012a; The MathWorks, Natick, MA) scripts were used to assimilate the results from Fraction CHECK and to display the data.

For each treatment parameter analyzed a correlation coefficient between the parameter values and the RMS error was determined. Correlation coefficients between ±0.5 and ±1.0 were considered strong. A p-value of <0.05 was taken to denote statistical significance. To determine whether institutions differed from one another in RMS error or 95^th^ percentile error, statistical analysis was done using analysis of variance between institutions within each treatment delivery type.

## Results

### Institutional comparison

**Figure 1:**
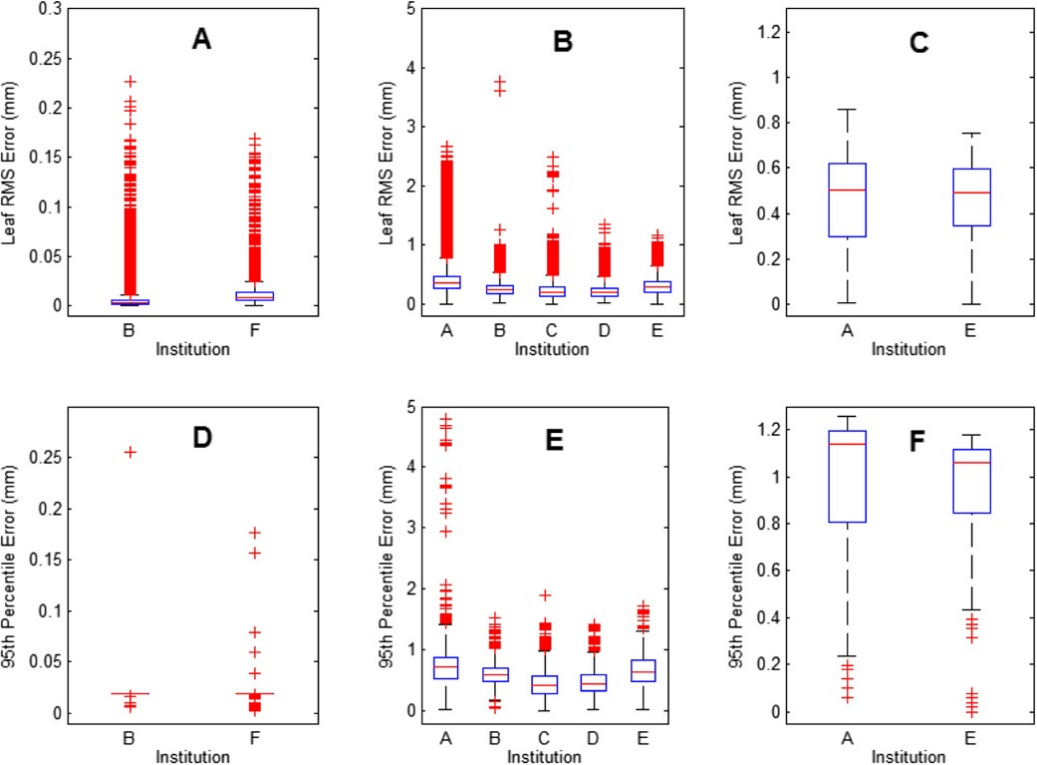
**Box plot of individual leaf RMS error values and 95** ^**th**^ **percentile values by treatment type. (A-C)** Leaf RMS error results plotted by institution for step & shoot, dynamic, and VMAT modalities respectively. **(D-F)** 95^th^ percentile errors plotted by institution for step & shoot, dynamic and VMAT modalities respectively. Results are plotted as normal boxplots with the top and bottom of the rectangle representing the 75^th^ (q3) and 25^th^ (q1) percentile values respectively and the median as the line within the box. The upper whisker corresponds to q3 + 1.5*(q3 – q1) and lower whisker to q1 – 1.5*(q3 – q1) or to the nearest data point thereof. Values above or below the respective whiskers are plotted as outliers.

Although TG-142 gives recommendations regarding the maximum leaf RMS error, we have rather presented the mean RMS leaf errors to provide a more representative summary of the data. The mean leaf RMS error and 95^th^ percentile error values were compared by institution and treatment type (Figure 1). The numbers of leaf RMS error data points by treatment modality were approximately 321,000 for step-and-shoot, 1,152,000 for dynamic, and 28,500 for VMAT. Table 2 shows the mean leaf RMS error and the mean 95^th^ percentile leaf error. The maximum RMS leaf errors are discussed in relation to TG-142 in the Discussion section. The step and shoot method had far lower leaf RMS error values than the other treatment types. The RMS error means of the dynamic and VMAT methods were similar, although there were many exceptions in the dynamic treatments but none were greater than 0.5 mm.

**Table 2:** **Summary of log file error results sorted by treatment type**

Modality	Institution	Mean leaf RMS error (mm)	Mean 95th percentile error (mm)
**Step & shoot**	B	0.004	0.020
	F	0.011	0.020
	Overall	0.008	0.020
**Dynamic**	A	0.37	0.69
	B	0.25	0.61
	C	0.22	0.45
	D	0.22	0.47
	E	0.30	0.65
	Overall	0.32	0.64
**VMAT**	A	0.46	0.96
	E	0.46	0.94
	Overall	0.46	0.95

Aggregated RMS error and 95^th^ percentile error values of each treatment type with values from contributing institutions and overall values listed.

With the exception of the VMAT data, the differences in average leaf RMS error between institutions were statistically significant. These differences may reflect differences in machine maintenance or plan difficulty. However, these differences were small, and are likely only significant because of the large volume of data analyzed (>1M points). For the step-and-shoot technique, the two institutions’ average leaf RMS error differed by 0.007 mm. For the dynamic method, the largest difference in mean leaf RMS error between any two institutions was 0.15 mm, and the largest difference in 95^th^ percentile error was 0.24 mm. These differences are unlikely to affect clinical dose delivery.

### Step-and-shoot

**Figure 2:**
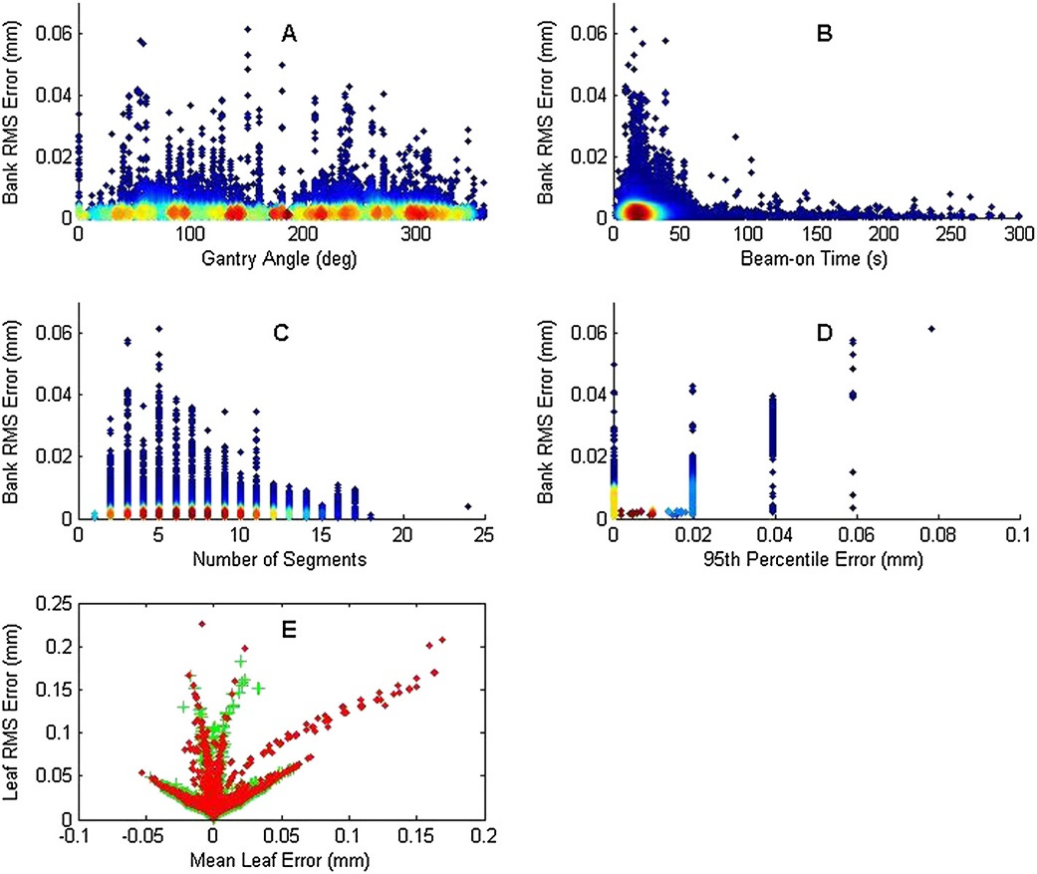
**Aggregated step and shoot treatment data plotting treatment parameters.** Individual leaf or bank RMS error is plotted according to **(A)** gantry angle, **(B)** beam-on time, **(C)** number of segments, **(D)** 95^th^ percentile error, **(E)** mean leaf error with each bank plotted separately. For **(A-D)**, data density is shown by color change; blue has low density while red has the highest density.

Leaf and bank RMS error were compared with several other mechanical treatment parameters for step-and-shoot IMRT (Figure 2). Figure 2(A) shows the bank RMS error as a function of gantry angle. A small sinusoidal pattern is suggested, with local maximums at angles of 90 and 270 and minimums near 0° and 180°, which could be theoretically explained by gravity. However, the data density shows that there is no substantial increase in RMS error, and thus the appearance of the increase in RMS error is due to outliers, which appear to be more prevalent at gantry angles near 90° and 270°. Bank RMS error did not correlate with total beam-on time (Figure 2[B]). The RMS error appears to peak initially for total beam-on times of 10-20 s and decrease thereafter as the beam-on time increases; however, this pattern is due to outliers as shown by the data density cloud. Bank RMS error also did not significantly correlate with the number of segments with no pattern discernible (Figure 2[C]); this stands in contrast to other investigations however [6].

Figure 2(D) plots the bank RMS error versus the 95^th^ percentile error count of the leaf deviations, which compares the two TG-142 parameters with each other. A strong correlation between these parameters would indicate that they are linked and therefore that only one parameter would be necessary to assess MLC performance. The parameters are in fact strongly and statistically correlated. This suggests that only one of these two parameters may be necessary to sufficiently analyze step-and-shoot MLC performance.

Figure 2(E) plots the individual leaf RMS error versus the mean leaf error for each bank. Since mean leaf error is signed it indicates the direction of the error. A mean leaf error of 0 indicates an even division between overshooting and undershooting the planned leaf position, while the opposite case, a linear correlation between RMS and mean leaf error would indicate a leaf steadily over or undershooting throughout the delivery. The mean leaf error data did not follow any specific pattern, being randomly distributed and having data evenly split on either side of 0 mm.

### Dynamic

**Figure 3:**
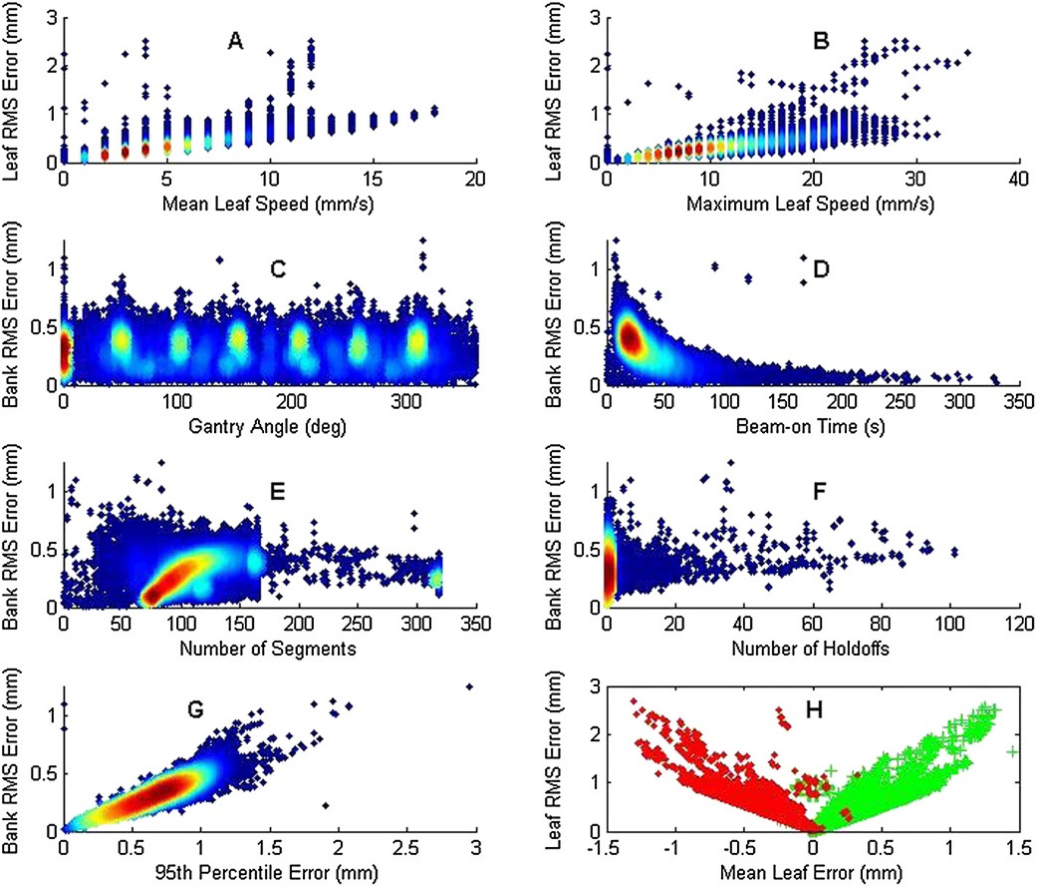
**Aggregated dynamic treatment data plotting treatment parameters.** Individual leaf or bank RMS error is plotted according to **(A)** mean leaf speed, **(B)** maximum leaf speed, **(C)** gantry angle, **(D)** beam-on time, **(E)** number of segments, **(F)** number of beam holdoffs, **(G)** 95^th^ percentile error, and **(H)** mean leaf error with each bank plotted separately. For **(A-G)**, data density is shown by color change; blue has little density while red has the highest density.

For the dynamic treatments, leaf speed is important because the leaves move while the beam is on; therefore, leaf speed was examined in addition to the parameters studied for step-and-shoot treatments (Figure 3). The individual leaf RMS error increased linearly with the mean leaf speed (Figure 3[A]) and with the maximum leaf speed (Figure 3[B]). Statistical analysis of the dynamic data showed a strong and significant correlation of the leaf RMS error with both mean leaf speed and maximum leaf speed. Because the maximum leaf speed can be controlled during treatment planning, this factor can be adjusted to control leaf RMS error.

The bank RMS error showed an even distribution across all gantry angles with no discernible pattern (Figure 3[C]) except to show that 0 is a common treatment angle. The bank RMS error can be seen to decrease with increasing beam time, and the cloud density shows this as well. In contrast to the step-and-shoot data, the beam-on time was determined to be significantly linked to the bank RMS error (Figure 3[D]). The number of field beam segments and the number of beam holdoffs due to an MLC being out of tolerance are shown in Figures 3(E) and (F), respectively. As with the step and shoot technique, the number of segments did not correlate with bank RMS error and neither did the number of beam holdoffs. The cloud density for the number of segments has an appearance of linearity between 75 and 125 segments, but the parameter is likely not significant because of the small cloud around 320 segments.

The bank RMS error correlated strongly and significantly with the 95^th^ percentile error (Figure 3[G]). These data suggest that when the mean RMS error of a treatment is high, the 95^th^ percentile error is very likely also high. This can be interpreted to mean that individual leaf performance does not vary substantially within the bank, otherwise the 95^th^ percentile error would show more spread amongst the data owing to underperforming leaves.

The individual leaf RMS error is plotted against the mean leaf error in Figure 3(H), with each bank plotted separately. The mean leaf error was split almost evenly in both directions, and correlated significantly with the leaf RMS error. The leaves of bank A consistently overshot the planned position across the treatment and thus had a positive mean leaf error, while bank B consistently undershot the planned position and thus had a negative mean leaf error. This type of systematic error may play a larger role dosimetrically than random errors, and large RMS errors may therefore be of particular concern for dynamic treatments.

#### VMAT

**Figure 4:**
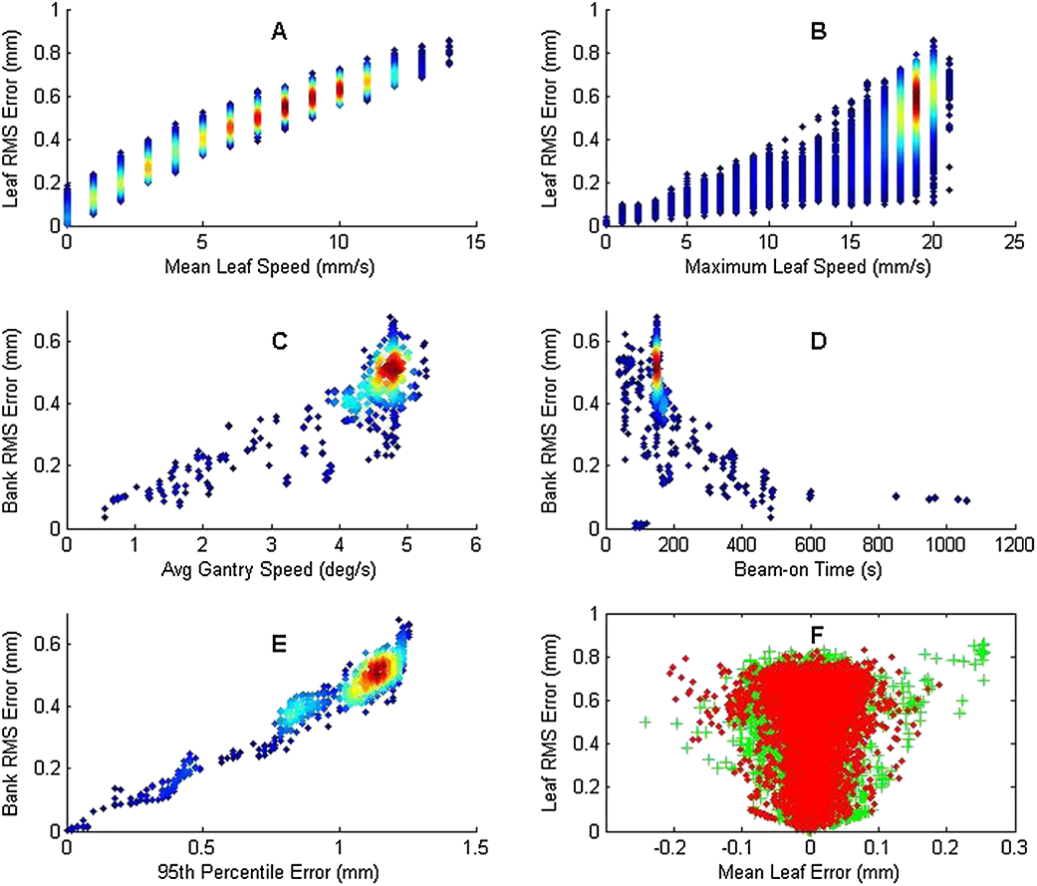
**Aggregated VMAT data plotting treatment parameters.** Individual leaf or bank RMS error are plotted according to **(A)** mean leaf speed, **(B)** maximum leaf speed, **(C)** average gantry speed throughout the treatment, **(D)** beam-on time, **(E)** 95^th^ percentile error, and **(F)** mean leaf error with each bank plotted separately. For **(A-E)**, data density is shown by color change; blue has low density while red has the highest density.

VMAT incorporates simultaneous gantry motion and dose rate variability along with leaf motion while the beam is on. Thus, parameters regarding gantry motion were among those examined for this modality. Figures 4(A) and (B) plot the leaf RMS error versus the mean leaf speed and versus the maximum leaf speed, respectively. As with the dynamic technique, there was a strong and significant correlation of the leaf RMS error with both the mean and maximum leaf speeds, agreeing with previous results [7]. The average of RMS error was higher for the VMAT treatments than for dynamic treatments, but the spread of VMAT leaf RMS error at each speed was also much narrower than that of the dynamic treatments.

The bank RMS error increased with increasing average gantry speed (calculated as arc span divided by beam time), although most speeds were 4°/s or greater (Figure 4C). Even so, bank RMS error significantly correlated with average gantry speed. The two VMAT institutions had relatively large differences in beam-on time (Figure 4D). For most VMAT deliveries at institution E, the beam-on time was 130-200 s, while most at institution A were 50-1000 s or more, indicating more partial arc treatments and possibly hypofractionated or stereotactic treatments. Despite these differences, the leaf RMS errors of both institutions were quite similar (Figure 1C), and the beam time was significantly correlated with the bank RMS, although most data lie around 150 seconds. For both average gantry speed and beam-on time, the large, tight clusters of data lessen the impact of the significant correlation of each.

Bank RMS error clearly and significantly correlated with the 95^th^ percentile error (Figure 4E). Just as for the dynamic deliveries, all leaves of a bank perform approximately the same otherwise the 95^th^ percentile error would be higher or have more variation.

Unlike that of the dynamic technique which showed a consistent correlation of leaves leading or lagging from their intended position, the distribution of mean leaf error for VMAT was clustered around 0 mm, and did not significantly correlate with the leaf RMS error (Figure 4F). These results indicate that the MLCs oscillated between overshooting and undershooting the planned position and that despite the fact that the leaf RMS error increased with the mean leaf speed, the net effect may have largely canceled out.

No holdoffs were issued by the clinac in any VMAT treatment. Therefore, this parameter was not studied.

## Discussion

Results of treatment logs from six institutions showed that RMS error and 95^th^ percentile leaf error were consistent between institutions, but varied by treatment type. On average, the step and shoot technique had very small errors, while VMAT had the largest errors, but only slightly more than the dynamic technique. MLC RMS error correlated with mechanical, treatment, and plan parameters. For the dynamic and VMAT techniques, the mean and maximum leaf speed was significantly linked to the leaf RMS error. For dynamic delivery, the mean leaf error correlated with leaf RMS error, whereas for VMAT the average gantry speed was also correlated. For all treatments, the RMS error and the 95^th^ percentile leaf error were correlated.

Parameters commonly thought to affect MLC performance were found to have no such effect including gantry angle (i.e. gravity), number of beam holdoffs, and number of segments. Similarly, treatments with numerous beam holdoffs would be expected to frequently approach the maximum error allowable as the leaves have reached tolerance, resulting in higher level of error overall, but this was not observed in the data. Finally, increases in leaf error due to an increasing number of field segments could be expected because a larger number of segments would be clinically associated with a larger number of small MU MLC shapes. However, the data for both step and shoot and dynamic delivery indicated that this parameter also did not affect the RMS error. Reasons for differences with previous studies may be an improvement in MLC controller performance and improved treatment planning algorithms.

Parameters that did affect MLC performance included the mean and maximum leaf speed, relevant to dynamic and VMAT treatments. As well, the dynamic data showed that the mean leaf error was systematically related to the RMS leaf error – leaves either systematically overshot or undershot their intended position. This relationship can be largely explained by the fact that dynamic treatments using Varian linacs and planned with the Eclipse treatment planning system move MLCs in only one direction. Another possible cause for this is lag between the MLC controller and the clinac [8]. If the lag observed is real, such a systematic leaf offset would result in a shift of the treatment fluence. This has the potential to have notable dosimetric effects as studies examining MLC shifts have shown that random shifts do not have a significant effect on dosimetry, whereas systematic shifts can [9–12]. Actual assessment of the dosimetric impact of the RMS errors seen here were not possible, and further study of this issue is warranted. Although specific recommendations are not given based on dosimetric outcomes, recommendations for better mechanical MLC performance can be made based on the present data.

As mentioned earlier, certain other parameters were not able to be analyzed, being both a strength and weakness. Dose rate in the clinical sense (600 MU/min, etc) is not recorded, but will affect the MLC speed and thus RMS error [13].

The recommendations of TG-142 are based largely on what the machine is able to do mechanically, not necessarily on its dosimetric impact and such are our recommendations here. Improved MLC performance can be most readily achieved by limiting the mean and/or maximum leaf speed. The machine dose rate and the internal tolerance were not able to be studied because it is either not recorded or did not vary in the log files, but they can have a significant effect on the performance. Restricting the internal tolerance of dynamic treatments to 1 mm limit the maximum leaf error, improving performance.

**Figure 5:**
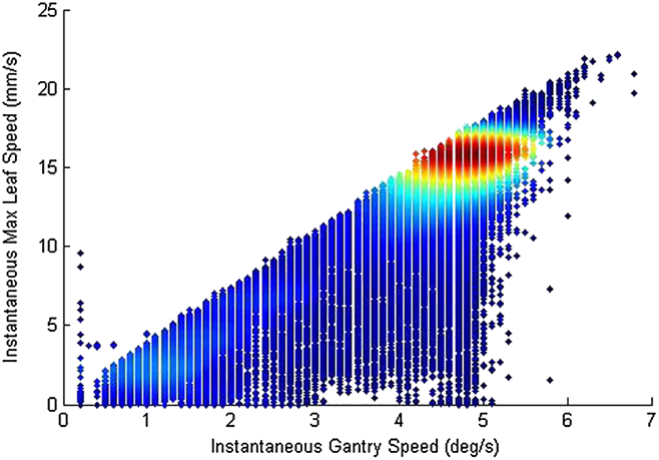
**In-depth analysis of VMAT logs.** Snapshots of 1 second intervals for every VMAT treatment log are plotted. Each snapshot/data point is the speed of the fastest leaf and the gantry speed.

For VMAT deliveries, in addition to the mean and maximum leaf speed, the average gantry speed was also shown to be correlated to bank RMS error. The reason for this relationship was not obvious and further study was done. Rather than average over the entire treatment, 1 s moving average window snapshots of the maximum leaf speed and the gantry speed were taken throughout each VMAT treatment (Figure 5; see Data Analysis for leaf speed calculations). The data show the strong and significant correlation between higher gantry speeds and higher maximum leaf speeds. The data density cloud shows that most data lay between 4 and 5 degrees/s, which are consistent with the data cluster of Figure 4(C). Thus, while there is a data cluster, it is still clearly shown that the maximum leaf speed and the gantry speed are correlated. Clinically, if the maximum leaf speed was limited to reduce the RMS leaf error, the gantry would slow down to accommodate the slower leaf travel to deliver a continuous plan [14]. Gantry speed variability was examined as a factor that could influence MLC RMS error, but no correlation was observed (data not shown). Figures 4(C) and 5 show that a slower gantry speed improves MLC delivery performance via a lower RMS error. So then, to improve VMAT treatments, we thus recommend lowering the maximum leaf speed, as was suggested for dynamic treatments, which would both improve MLC performance and potentially force down the maximum and average gantry speed as it depends on the MLC distance per degree limit [14]. The dosimetric effect of errors due to high gantry speed is unknown, but is likely to be small for VMAT treatments [10]. This does not negate the RMS error however.

Evaluation of the RMS error and 95^th^ percentile error, for which TG-142 recommends tolerances of 3.5 mm, is critical to understanding the current performance level of MLCs. Noticeably, almost all data in Figure 1 are well below TG-142 criteria. In fact, only a handful (out of the ~85,000) in the dynamic modality are above the TG-142 tolerance value. Therefore, based on current performance, it would be prudent to implement more appropriate criteria for Varian machines.

Table 3 shows percentages of leaf RMS errors that fail according to different tolerance levels for the data examined in the present study. The error with the step and shoot technique was so low (Table 2) that even with 0.5 mm tolerances, no data were ever outside of criteria. Similarly, for VMAT, no data were ever beyond 1.5 mm. Dynamic delivery was the only technique where larger errors were observed, but only 0.03% of dynalog files showed an RMS error greater than 2 mm, and 0.06% of files showed a 95^th^ percentile error greater than 2 mm. These data suggest that the TG-142 criteria may be unnecessarily loose for Varian machines.

**Table 3:** **Log failure rates with various tolerances applied**

	Leaf RMS error					95th percentile error				
Tolerance (mm)	0.5	1.0	1.5	2.0	3.5	0.5	1.0	1.5	2.0	3.5
				**Log failure %**						
Step & shoot	0	0	0	0	0	0	0	0	0	0
Dynamic	14.02	0.12	0.06	0.03	0	67.98	8.16	0.13	0.06	0.05
VMAT	49.30	0	0	0	0	85.75	61.44	0	0	0

Failure percentages of the data presented in this study for various proposed thresholds of the two metrics in TG-142 for Varian machines. The current TG-142 thresholds of 3.5 mm are shown for comparison.

In clinical practice, the leaf error results of MLC tests can differ greatly from test to test and from modality to modality, depending on the nature of the test, treatment site, plan complexity, and the average and/or maximum leaf speeds and should be accounted for when performing, for example, demanding MLC QA tests or complex treatment plans or when introducing a new treatment technique into the clinic. Because of this, the MLC RMS error of a strenuous test should not be assumed as what a typical treatment RMS error is. However, on the basis of the results of Table 3, a tolerance value of 1 mm is likely an appropriate criterion for the maximum RMS leaf error tolerance or action level, of all treatment techniques. Correspondingly, the 95^th^ percentile error threshold should likely be 1.5 mm. MLC leaf position errors above such a threshold do not necessarily indicate a dosimetric or calibration problem, but could indicate a difficult plan to deliver, pushing the MLCs to their limits, and can therefore serve as possible action levels.

Another point of interest is the correlation between bank RMS error and the 95^th^ percentile error. All treatment techniques were significantly correlated and generally had little spread in the data. It can be concluded from the results that a high leaf RMS error will not be unnoticed via the 95^th^ percentile error and vic versa, suggesting that only one metric may be needed for this part of MLC QA. What is also noteworthy about the correlation is the very comparable performance of MLCs, even among different institutions, presumably with different QA standards. Dirt and grease can affect performance, but the data presented here shows consistent performance [15]. It would therefore be unusual for one leaf to be noticeably slower than the rest of the MLC bank. A discrepancy between the expected values of the 95^th^ percentile error and the bank RMS would quickly reveal such a leaf rather than having to visually inspect the leaves as they move during tests (e.g. the picket fence test).

The number of metrics a physicist can use depends on the analytic power of the software they use. The most basic way of assessing the logs is through the Dynalog File Viewer, which explicitly gives the mean and maximum RMS error of the leaves and shows a graph of the leaf errors in bins, but does not explicitly give the 95^th^ percentile error. Homemade scripts can be made to assess the log files automatically and can show additional metrics; a graphical user interface is also available from the authors. Furthermore, commercial software as used in this study also shows more metrics and can be used for trend analysis.

## Conclusion

This study examined MLC log data from Varian linacs, specifically dynalogs, to determine typical MLC performance during treatments, which mechanical treatment factors influence MLC leaf error, and whether the tolerance values of MLC performance metrics given in TG-142 are appropriate. Between institutions, the typical leaf RMS error values and 95^th^ percentile error values were not substantially different from each other. Comparing treatment modalities, step and shoot had the lowest typical leaf RMS error at 0.008 mm, dynamic treatments had a value of 0.32 mm, and VMAT had 0.46 mm.

Most other mechanical treatment parameters, like gantry angle, number of beam holds, and number of segments did not correlate to an increase in RMS error. For both dynamic and VMAT treatments, the parameters found to affect leaf RMS error were mean and maximum leaf speed. Furthermore, for dynamic treatments the mean leaf error was also correlated. For VMAT treatments, the average gantry speed was also found to correlate with RMS error. Considering that physicists can usually adjust the maximum leaf speed parameter within their treatment planning system, reducing leaf RMS error is reasonably achievable if need be and would positively affect both dynamic and VMAT deliveries.

In examining the MLC performance and distribution of error in a large number of treatments across multiple clinics, we recommend for Varian machines to use tighter tolerances than TG-142 gives, as those metric values appear to be inappropriately loose compared to physical machine performance. Various tolerance values or action levels were derived from the large dataset (Table 3) to reflect real-world performance. A tolerance value of 1 mm is recommended either as the maximum leaf RMS error or an action level for investigation for all treatment techniques and 1.5 mm is recommended for the 95^th^ percentile error.

MLC logs continue to be a valuable piece of information to study to better understand individual treatments as well as assess overall performance. Additional treatment data will shed more light on the accuracy of these recommendations.
